# Hyperspectral imaging reveals that sapropelic mud therapy may improve local tissue oxygenation in elderly

**DOI:** 10.1007/s00484-024-02838-8

**Published:** 2024-12-05

**Authors:** Mihaela Antonina Calin, Dragos Manea, Sorin Viorel Parasca, Cristina Popescu, Elena-Valentina Ionescu, Constantin Munteanu

**Affiliations:** 1 National Institute of Research and Development for Optoelectronics INOE 2000, 409 Atomistilor Street, P.O. BOX MG5, Magurele, Ilfov, 077125 Romania; 2 Carol Davila University of Medicine and Pharmacy, 37 Dionisie Lupu Street, Bucharest, Romania; 3 Neuromuscular Rehabilitation Division, Clinical Emergency Hospital “Bagdasar-Arseni”, Bucharest, 041915 Romania; 4 Balneal and Rehabilitation Sanatorium of Techirghiol, Techirghiol, 906100 Romania; 5 Department of Biomedical Sciences, Faculty of Medical Bioengineering, University of Medicine and Pharmacy “Grigore T. Popa”, Iași, Romania

**Keywords:** Oxyhemoglobin, Deoxyhemoglobin, Oxygen saturation, Neuromuscular disorders, Hyperspectral imaging

## Abstract

Sapropelic muds have been used for centuries to treat various illnesses, but their effects and mechanisms are still under research. In this study the effects of Techirghiol sapropelic mud on tissue oxygenation in elderly patients diagnosed with neuromuscular disorders were investigated using spatial and spectral information provided by hyperspectral imaging technique. A group of 38 elderly patients with neuromuscular disorders for which they received mud therapy was studied. Sapropelic mud was applied to the lumbar region of each patient for 30 min, directly on the skin in a thick layer, while a symmetrical area of ​​15 × 10 cm in the same region was covered with a medical patch to serve as the control area. The mud is typically heated to a temperature of 40–45 °C before application. Hyperspectral images were taken before, after the first day of therapy, and at day seven. Oxyhemoglobin and deoxyhemoglobin concentrations and oxygen saturation values were calculated from the hyperspectral images and compared to control areas. The results revealed that, in the treated area, the mean oxyhemoglobin concentration increased with + 0.2127 ± 0.1096 mol cm∕L, while deoxyhemoglobin concentration decreased by -0.0509 ± 0.0558 mol cm∕L. Local tissue oxygen saturation raised to over 98% in all patients. Lesser improvements were recorded for the control areas: oxyhemoglobin increased with + 0.1673 ± 0.1059 mol cm∕L, and deoxyhemoglobin decreased with − 0.0525 ± 0.0578 mol cm∕L. A good level of agreement was found between values of oxygen saturation measured with hyperspectral imaging method and the classical pulse oximetry method. Thus, improvement in local circulation was demonstrated after mud therapy. In conclusion, therapy with Techirghiol sapropelic mud improved local tissue oxygenation, hyperspectral imaging being a reliable and non-invasive tool for monitoring these changes.

## Introduction

Mud therapy, also known as pelotherapy, has been used for centuries as a natural therapeutic approach for treating diseases and disorders. It involves the external application of therapeutic mud, which is rich in organic and inorganic components, to improve health through both thermal and chemical effects. Over time, therapeutic mud has been found to have beneficial applications in several medical fields, particularly in the treatment of various rheumatological disorders (Mingrone et al. 2020; Gouvêa et al. 2021; Tognolo et al. 2022; Boopalan et al. 2024), dermatological disorders (Comacchi and Hercogova 2004; Costantino and Lampa 2005; Supriya and Maheswari 2015; Mingrone et al. 2020), cardiovascular disorders (Costantino et al. 2015; Valsakumar et al. 2022), gynecological disorders (Min et al. 2020; Habek et al. 2021) and neurological (Khoo et al. 2018; Filatova et al. 2018) disorders. Its efficacy is thought to be derived from its ability to influence physiological processes at the cellular and tissue level through thermal, mechanical, and chemical actions.

Therapeutic mud is a complex mixture composed of clay minerals and organic matter combined with mineral water, resulting from a maturation process (Gomes et al. 2013). The composition and concentration of inorganic and organic components, as well as the physical properties of the mixture (density, plasticity, texture, specific heat capacity, thermal conductivity, viscosity, etc.) distinguish among different types of mud and their therapeutic effects. For instance, Techirghiol mud, classified as a sapropelic peloid, is harvested from Lake Techirghiol located in Romania, in the Northern Dobrogea region near the town with the same name and it is known for its high organic content, rich mineral composition, and therapeutic properties (Cadar et al. 2021). This mud has demonstrated beneficial effects in the treatment of different pathologies including osteoarthritis (Ionescu et al. 2017; Demirgian et al. 2018; Iliescu et al. 2022), low back pain (Tica et al. 2019), rheumatic diseases (Teleki N. 1984), sarcopenia (Stanciu et al. 2023), and other conditions including chronic eczema, psoriasis, and wounds (Ciobotaru et al. 2009).

Despite the extensive use of therapeutic muds, much remains to be understood about their underlying mechanisms, particularly their effects on blood flow, lymphatic circulation, and tissue oxygenation. Previous studies have suggested that mud applications can improve microcirculation and stimulate vasomotion. For example, Poensin et al. (Poensin et al. 2003) reported a significant increase in blood flow and stimulation of vasomotion after the application of „La Léchère” mud pack (France) in patients diagnosed with lower limb venous insufficiency and osteoarthritis, as measured by laser-Doppler flowmetry. A similar effect was also reported by Clijsen et al. (Clijsen et al. 2008) when applying mud packs (“Parafango di Battaglia”, Padova, Italy) to healthy subjects. Their results showed that skin micro-perfusion measured by Laser-Doppler flowmetry increased significantly during mud pack application, from 23.2 ± 8.8 to 197 ± 41 p.uMore recent research has shown that sulfur-rich silt mud from Lake Karantinnoe improved facial skin microcirculation in women (Kasimova et al. 2017). In a study conducted by Tuulik et al. (Tuulik et al. 2015) on upper limbs perfusion in case of the professional overuse, therapeutic mud was shown to restore microcirculation in upper extremity overuse syndrome with moderate pain, with significant changes in perfusion, rest flow, and peak flow being reported. Promising results were also reported by Marin et al. (Marin et al. 2011, 2012) who evaluated the impact of sapropelic mud from Lake Techirghiol on peripheral circulation based on blood samples analysis collected from patients on the first day and at the end of treatment. An improvement in peripheral vascularization after mud therapy was reported and demonstrated by statistically significant changes in the partial blood gases pressure. However, these studies have largely focused on general circulatory effects, and there is limited research on the specific impact of mud therapy on tissue oxygenation.

The present study aims to address this gap by investigating the effects of Techirghiol sapropelic mud on tissue oxygenation in elderly patients diagnosed with neuromuscular disorders. To achieve this, hyperspectral imaging (HSI), a non-invasive technique that captures both spatial and spectral information from biological tissues was used. First introduced by Goetz et al.(Goetz et al. 1985) in the late 1980s for Earth observation, hyperspectral imaging has now also found applications in the medical field due to its ability provide detailed insights on the structure and chemical composition of biological tissues at different levels of scientific investigation.

In this study, hyperspectral imaging combined with an analytical model based on the modified Beer-Lambert law was used to generate concentration distribution maps of the main chromophores of the skin, especially oxyhemoglobin and deoxyhemoglobin. These distribution maps allow for the precise monitoring of changes in tissue oxygenation and help visualize the effects of mud therapy. By leveraging this advanced imaging technique, the study aims to provide new insights into the role of Techirghiol sapropelic mud in improving local circulation and tissue oxygenation, thereby enhancing our understanding of its clinical benefits.

## Materials and methods

### Patients

This research was designed as an observational, non-randomized, controlled (each patient was their own control) study aimed at evaluating the therapeutic effects of Techirghiol sapropelic mud applications on local tissue oxygenation in elderly patients with neuromuscular disorders. The primary outcome was to assess tissue oxygenation changes using quantitative hyperspectral imaging. The study adhered to ethical standards, and approval for the protocol was obtained from the Ethics Committee of the Techirghiol Balneary and Rehabilitation Sanatorium (No. 5232/20.01.2024). All procedures involving human participants were performed in accordance with the 1964 Helsinki Declaration and its subsequent amendments or comparable ethical standards.

A total of 38 elderly patients (9 men and 29 women), aged between 53 and 85 years, diagnosed with various neuromuscular disorders, hospitalized in the Techirghiol Balneary and Rehabilitation Sanatorium during January 2024 were enrolled in this study (Table 1).

**Table 1:** Subjects’ characteristics

Characteristics	Values
Number of subjects	38
Age	
Mean (± SD), years	67.58 ± 10.25
Range, years	63–85
Sex, n(%)	
Male	9 (23.68)
Female	29 (76.32)
Pathology, n (%)	
lumbar discopathy	11 (28.95%)
generalized osteoarthritis	9 (23.68%)
lumbar spondylodyscartrosis	18 (47.37%)

None of the patients selected in the study had acute or decompensated conditions or diagnoses of cancer, cardiopulmonary, or vascular disorders, and did not have surgery in the last three months.

### Mud therapy

Techirghiol sapropelic mud was applied to the lumbar area of each patient (Study Area - SA) as part of the daily mud therapy, which lasted for 7 days. The mud was directly applied to the skin, with the exception of a 15 × 10 cm area (Control Area – CA) in the left lumbar region, which was covered with a medical patch and designated as the control area. The medical patch served to protect the control area from contact with the mud, allowing for a comparative analysis of the effect of the mud on tissue oxygenation. The control area was standardized and consistently applied in the same region for all patients, ensuring that the treated and control areas were anatomically comparable.

Before application, the Techirghiol mud was heated to a temperature of approximately 40 °C to 45 °C to enhance its therapeutic properties. This temperature range promotes muscle relaxation and vasodilation, which are essential for improving blood circulation in the treated area. Each patient’s lumbar region was covered with an uniform layer of mud, approximately 2 to 3 cm thick. The treated area (SA), excluding the 15 × 10 cm control area (CA), covered an area of approximately 30 × 40 cm, ensuring consistent treatment across participants. The mud was applied for 30 min per session. During this time, patients were kept in a comfortable, supine position to allow for the optimal effect of the mud. The medical patch used to cover the 15 × 10 cm control area (CA) was placed within the lumbar region, in a symmetrical zone, ensuring that no mud made contact with it. The patch was applied only during the mud application, as occlusion of the skin (with the patch) could stimulate vasodilation, potentially confounding the results. Following each mud therapy session, patients were given a cleansing shower with tap water to remove any residual mud. No mineral or therapeutic water was used during the cleansing process. Throughout the treatment period, all patients continued their prescribed medications without interruption, and no additional therapies, such as thermal water baths, were administered during the course of the study. To ensure consistency, all applications were performed by trained personnel using the same method and tools. To maintain uniformity in the treatment protocol, each patient’s selection of the control area and the treated area was standardized in the study protocol. The consistency of the mud, its heating process, and the thickness of the applied layer were maintained according to the predefined protocol.

### Hyperspectral image acquisition and processing

**Fig. 1:**
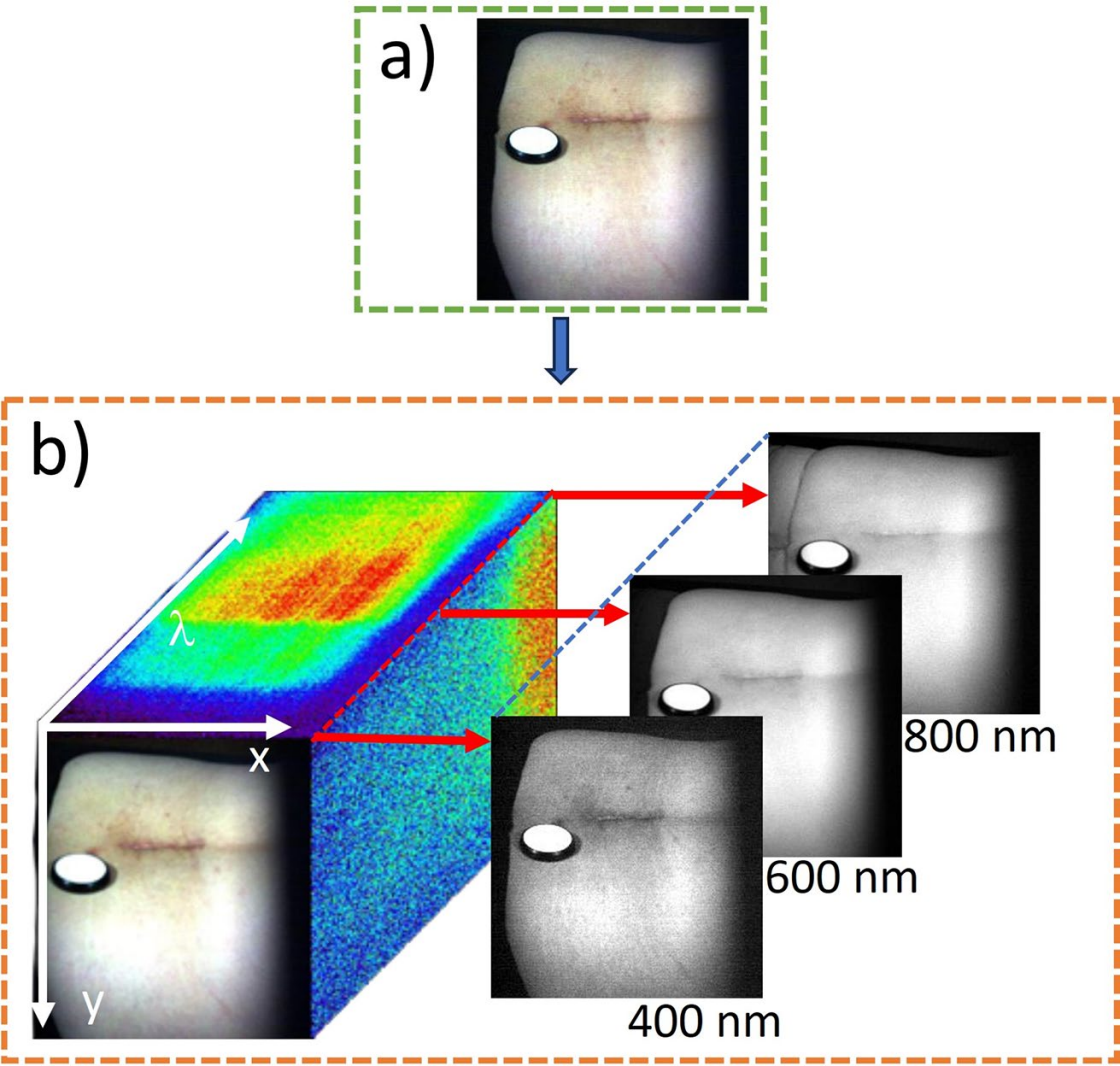
Hyperspectral image of a 68-year-old patient diagnosed with low back pain, acquired using the line-scan hyperspectral imaging system. (**a**) hyperspectral image of the patient’s lumbar area, (**b**) hypercube consisting of 205 spectral bands in the (400–800) nm spectral range, with examples at spectral bands 400 nm, 600 nm, and 800 nm

Hyperspectral images of the investigated areas (with and without mud applied), each consisting of 205 adjacent narrow spectral bands (called hypercube, Fig. 1), were acquired before mud therapy, after the first day of treatment and at the end of mud therapy, with the patients sitting on their ventral side in a relaxed position, using a line-scan hyperspectral imaging system (ImSpector V8E, Specim, Oulu, Finland) in a (400–800) nm spectral range at a spectral resolution of 1.97 nm and a frame rate of 41 fps. Illumination of the investigated areas was provided by an illumination unit consisting of two 300 W halogen lamps (OSRAM, Munich, Germany) with diffusion filters (Kaiser Fototechnik GmbH and Co. KG, Buchen, Germany) fixed above the lumbar area of the patients on both sides, at an angle of approximately 45º to ensure uniform illumination of the area of interest. A single-axis galvanometer scanning mirror system (GVS211, Thorlabs, New Jersey, USA) equipped with a broadband dielectric mirror was used for line-by-line scanning of the investigated areas. The hyperspectral image acquisition process was controlled by a computer using SpectralDAQ data acquisition software (Specim, Oulu, Finland). Subsequent processing and analysis of hyperspectral images was performed using ENVI v.6 software (Exelis Visual Information Solutions, Boulder, Colorado, USA).

**Fig. 2:**
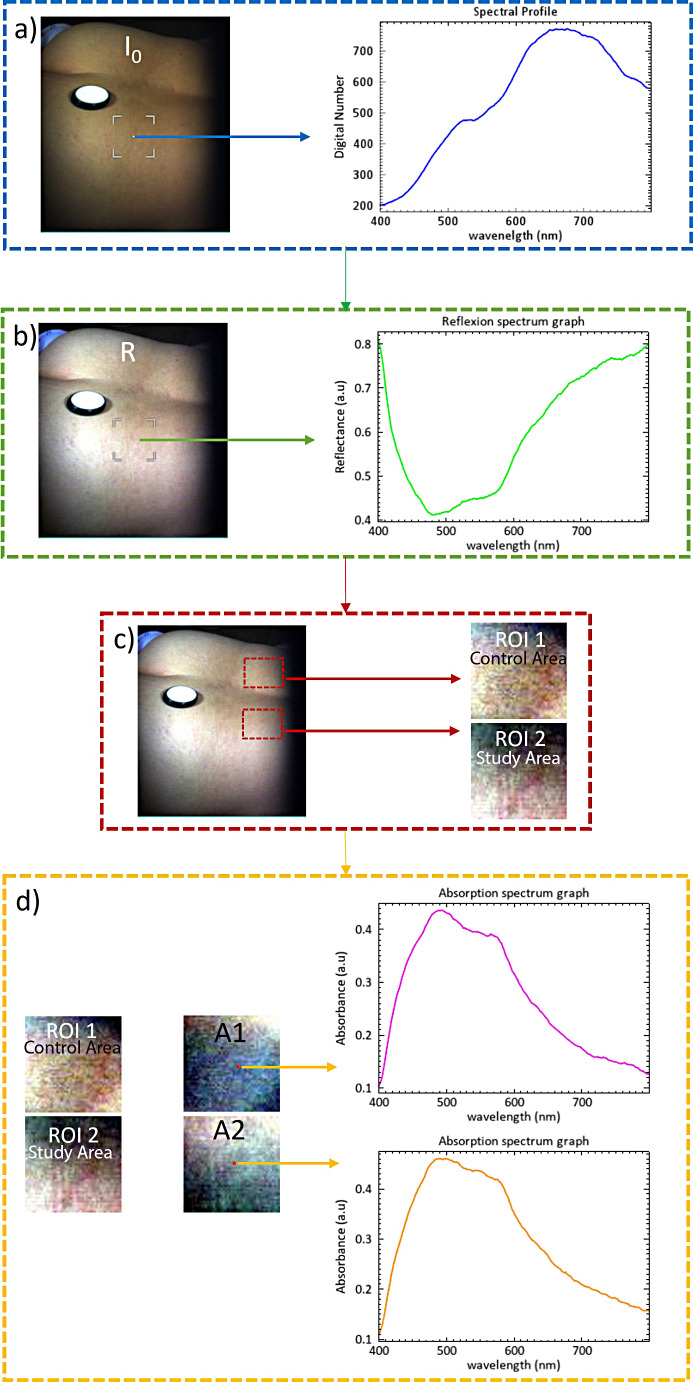
Flowchart illustrating the stepwise processing of hyperspectral images of the investigated areas. (**a**) original hyperspectral image (I_o_) and corresponding spectral profile (DN(λ)); (**b**) calibration of the original hyperspectral image resulting in a reflectance image (R) and illustration of the corresponding reflectance spectrum (R(λ)); (**c**) selection of regions of interest (ROIs) (study (ROI 1) and control (ROI 2)); (**d**) conversion of reflectance images (ROIs) to absorbance images (A) and their corresponding absorbance spectra (A(λ))

The hyperspectral images thus acquired contain data relating to the intensity values of the pixels expressed by a digital number (DN). In order to be able to extract relevant information from these hyperspectral data, the pixel values must be converted into physical quantities, such as reflectance, which is in correlation with the principle of hyperspectral imaging. This conversion was performed as a first step in the processing of the original hyperspectral images (Fig. 2). The conversion process, also called calibration process, was achieved by using black and white reference hyperspectral images and calculating the reflectance values of each pixel in the image according to Eq. 1:

1 $$\:R=\frac{{I}_{O\:}-{I}_{D}}{{I}_{W}-{I}_{D}}$$

where: R is the calibrated reflectance image (Fig. 2b), I_O_ is the original hyperspectral image (Fig. 2a), I_D_ and I_W_ are the dark and white reference image, respectively. The dark reference image (I_D_) was acquired by covering the lens of the HSI system with its black cap. The white reference image (I_W_) was acquired from a polytetrafluoroethylene (PTFE) standard white reference tile (model WS-2, Avantes, Apeldoorn, Netherlands) with approximately 98% reflectance in spectral range 350–1800 nm, located in the investigated scene (Fig. 2a).

Figure 2.

The calibrated reflectance images thus obtained contain some information irrelevant for the purpose of this study that may influence the accuracy of the data analysis results. Consequently, irrelevant elements in each reflectance image were removed, in the second stage of image processing, by a manual selection of two regions of interest (ROI) covering the lumbar region (ROI 1- study area) or patch area (ROI 2 - control area) (Fig. 2c).

Considering that any mud therapy-induced change in the chemical composition of biological tissues is reflected in the absorption spectra of their components, a conversion of the calibrated reflectance images (ROIs) to absorbance images (A) was further performed using Eq. (2):

2 $$\:A=-\text{log}R$$

The resulting ROI absorbance images (Fig. 2d) were used for subsequent chemometric analysis of skin chromophores.

### Hyperspectral image analysis

The analysis of ROI absorbance images was performed using an analytical model previously developed by us (Miclos et al. 2015) to calculate the distributions of oxyhemoglobin (HbO_2_) and deoxyhemoglobin (Hb) concentrations in the skin. The model unmixes the contributions of these two main skin chromophores (HbO_2_ and Hb), which can be used for oxygenation assessment, to the estimated absorbance A_S_(λ) of each pixel (i, j) in the ROI absorbance image according to the modified Beer–Lambert law (Sassaroli and Fantini 2004), using Eq. (3):

3 $${A_S}\left(\lambda \right) = {\varepsilon _{HbO2}}\left(\lambda \right)C_{HbO2}^S + {\varepsilon _{Hb}}\left(\lambda \right)C_{Hb}^S + G$$

where: A_S_(λ) is the estimated absorbance spectrum in pixel (i, j) in the ROI absorbance image, λ represents the wavelength of light, ε_HbO2_(λ) and ε_Hb_(λ) are the extinction coefficients (cm^− 1^/(mol/liter)) for oxyhemoglobin and deoxyhemoglobin, respectively, $\:{C}_{HbO2}^{S}$ and $\:{C}_{Hb}^{S}$ represent the surface molar concentrations of oxyhemoglobin and deoxyhemoglobin [(mol∕L) cm] at the pixel (i, j) defined as C^S^ = C L, L is the mean path of a photon within the tissue (cm), and G is a term that includes the light scattering effect in each pixel (i, j) in the image.

Nonlinear least square analysis was used to fit the calculated absorbance data (Eq. 2) in each pixel (i, j) in the ROI absorbance image to the estimated absorbance data described by the model (Eq. 5) and to find the set of parameters $$\:{C}_{HbO2}^{S},$$$$\:{C}_{Hb}^{S}$$, and G in each pixel (i, j) for which the calculated data fits best the estimated data in the least squares sense, i.e. the sum-of-squares difference expressed by Eq. 4 is minimized.

4 $$\mathop \sum \limits_{k = 1}^n {\left\{ \begin{gathered} A\left({_k} \right) - \hfill \\\,\left[ \begin{gathered}{\varepsilon _{HbO2}}\left({_k} \right){C_{HbO2}} \hfill \\+ \,{\varepsilon _{Hb}}\left({_k} \right){C_{Hb}} + G \hfill \\ \end{gathered} \right] \hfill \\ \end{gathered} \right\}^2} \to min$$

where: λ_k_ are the wavelength values at which absorbance data are calculated in the spectral range (400–800) nm, k takes values from 1 to n, and n represents the total number of spectral bands (*n* = 205).

The Levenberg–Marquardt algorithm (LMA), also known as the damped least-squares (DLS) method, was used to solve non-linear least squares minimization problem. Since the LMA algorithm required an initial guess for the parameters to be estimated, this was done after several tests and a set of initial values for the specified parameters was established as follows: $\:{\text{C}}_{\text{H}\text{b}\text{O}2}^{\text{S}}=5\:\text{m}\text{o}\text{l}\cdot\:\text{c}\text{m}/\text{L}$, $\:{\text{C}}_{\text{H}\text{b}}^{\text{S}}=5\:\text{m}\text{o}\text{l}\cdot\:\text{c}\text{m}/\text{L}$, and G = max(A-1). The solutions thus obtained to the minimization problem are distribution maps of the surface molar concentrations of HbO_2_ and Hb displayed as color-coded images.

In addition, from these oxyhemoglobin and deoxyhemoglobin molar surface concentration distribution maps, oxygen saturation (StO_2_) distribution maps can also be obtained, using Eq. 5:

5 $$\:{StO}_{2}=\frac{{C}_{HbO2}^{S}}{{C}_{HbO2}^{S}+\:{C}_{Hb}^{S}}\:\cdot\:\:100\:$$

These distribution maps allow easy visualization and analysis of any mud therapy-induced changes in skin oxygenation status and can support physicians in non-invasive and low-cost monitoring of treatment effects.

### Pulse oximetry measurements

Pulse oximetry measurements of the index finger were performed in all patients for the non-invasive measurement of blood oxygen saturation in order to compare and validate the results of the proposed model. Blood oxygen saturation (StO_2_) was measured using a pulse oximeter (P02, Easypix, Köln, Germany) simultaneously with the acquisition of hyperspectral images, under the same pressure and temperature conditions (same experimental room).

### Statistical analysis

The performance of the nonlinear regression model was assessed using the coefficient of determination R^2^ which indicates how well-calculated absorbance values for each pixel in the image are estimated by the model. It is mathematically defined by Eq. 6:

6 $$\:{R}^{2}=1-\:\frac{\sum\:_{k}^{n}{\left[A\left({}_{k}\right)-{A}_{S}\left({}_{k}\right)\right]}^{2}}{\sum\:_{k}^{n}{\left[A\left({}_{k}\right)-\:\frac{\sum\:_{k}^{n}A\left({}_{k}\right)}{n}\right]}^{2}}$$

where: A(λ_k_) represents the calculated absorbance values at wavelength λ_k_ for a pixel (i, j) according to Eq. 2, A_S_(λ_k_) represents the values estimated by the model for the absorbance at the wavelength λ_k_ for a pixel (i, j), according to Eq. 3 and n is the number of wavelengths (spectral bands) considered. The values of R^2^ range from 0 to 1. R^2^ values close to 1 indicate that the estimated absorbance values are accurate, while R^2^ values close to 0 indicate that the model fails to accurately estimate the data.

The results were expressed as mean ± standard deviation. The Shapiro-Wilk test was used to check the normality of the data. Intra-group differences (before, after one day, and at the end of mud therapy) were assessed using a paired-samples t-test. Inter-group differences (at the same time points of mud therapy) were also performed using paired-samples t-test. A p-value of less than 0.05 was considered for statistical significance. The degree of agreement between the oxygen saturation (StO_2_) values calculated from the hyperspectral data and those measured by pulse oximetry was assessed using the statistical method proposed by Bland and Altman (Bland and Altman 1986). This statistical method quantifies the mean difference between StO_2_ values determined by the two methods using a graphical method consisting of a scatter plot where the difference of paired values is plotted against the mean of the two measurements, with two lines ΔStO_2med_ ± 2 standard deviations parallel to the mean difference line (which assures a 95% confidence interval). All statistical analyses were performed using SPSS software v23 (International Business Machines Corporation (IBM), New York, United States) and ORIGIN v 9.75 (OriginLab Corporation, Northampton, United States).

## Results

**Fig. 3:**
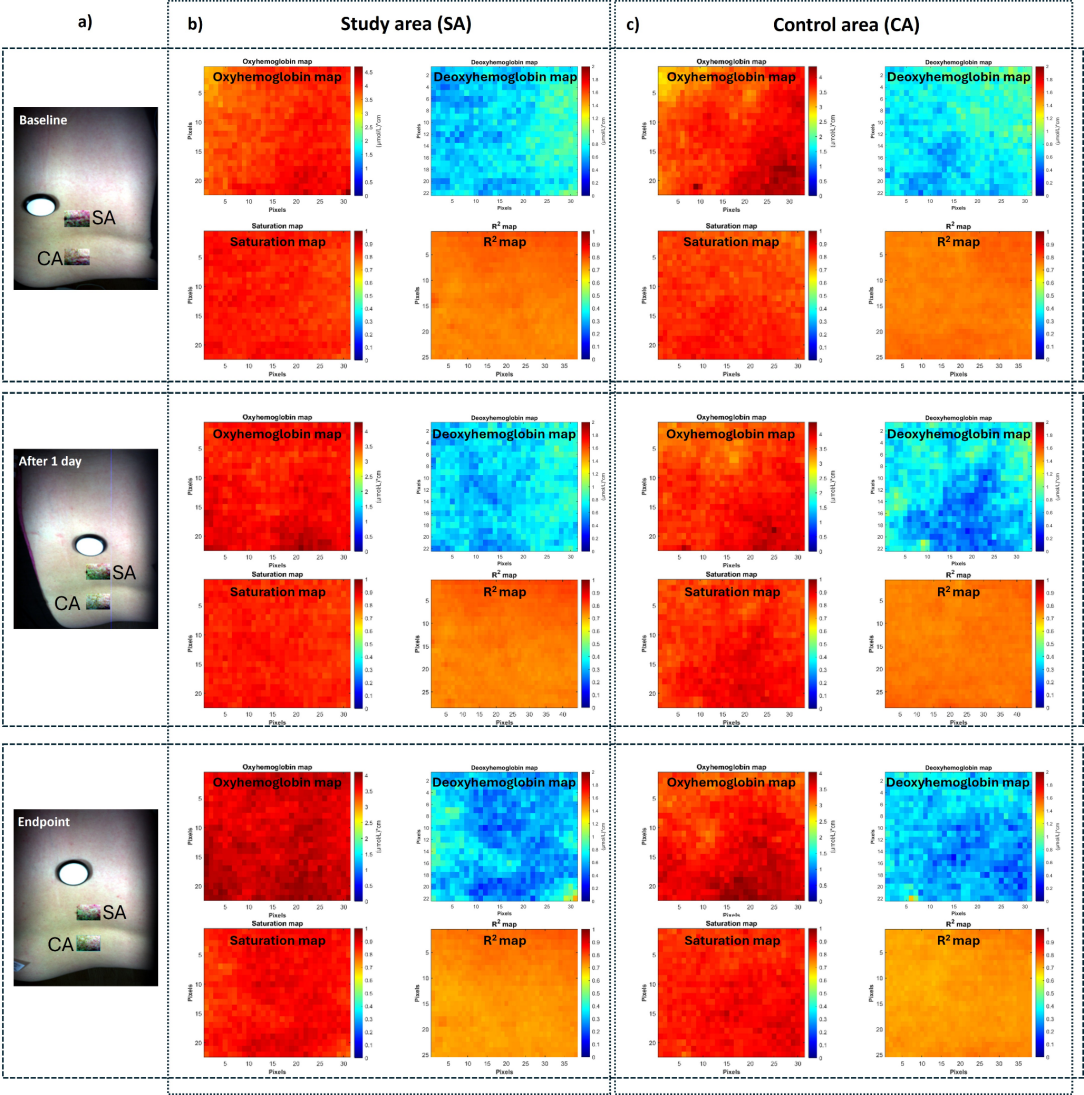
Distribution maps of concentrations of oxyhemoglobin and deoxyhemoglobin, oxygen saturation (StO_2_) and coefficient of determination (R^2^) calculated from the ROIs related to the two investigated areas on the lumbar area of a 66-year-old patient diagnosed with low back pain. (**a**) hyperspectral images of the patient’s back at different time points during the mud therapy (baseline, after 1 day of treatment and at the end of the treatment) illustrating the investigated areas; (**b**) maps showing the distributions of concentrations of HbO_2_, Hb, StO_2_ and R^2^ on the investigated study area at different time points during mud therapy; (**c**) maps showing the distributions of the concentrations of HbO_2_, Hb, StO_2_ and R^2^ on the investigated control area at the same time points during mud therapy

Figure 3 shows the distribution maps of the concentrations of oxyhemoglobin and deoxyhemoglobin, as well as the distribution maps of the oxygen saturation (StO_2_) and the coefficient of determination (R^2^) on the ROIs related to the two investigated areas on the lumbar area of one patient (SA - mud treated area and CA - untreated area) generated from hyperspectral images acquired at different time points during mud therapy.

**Fig. 4:**
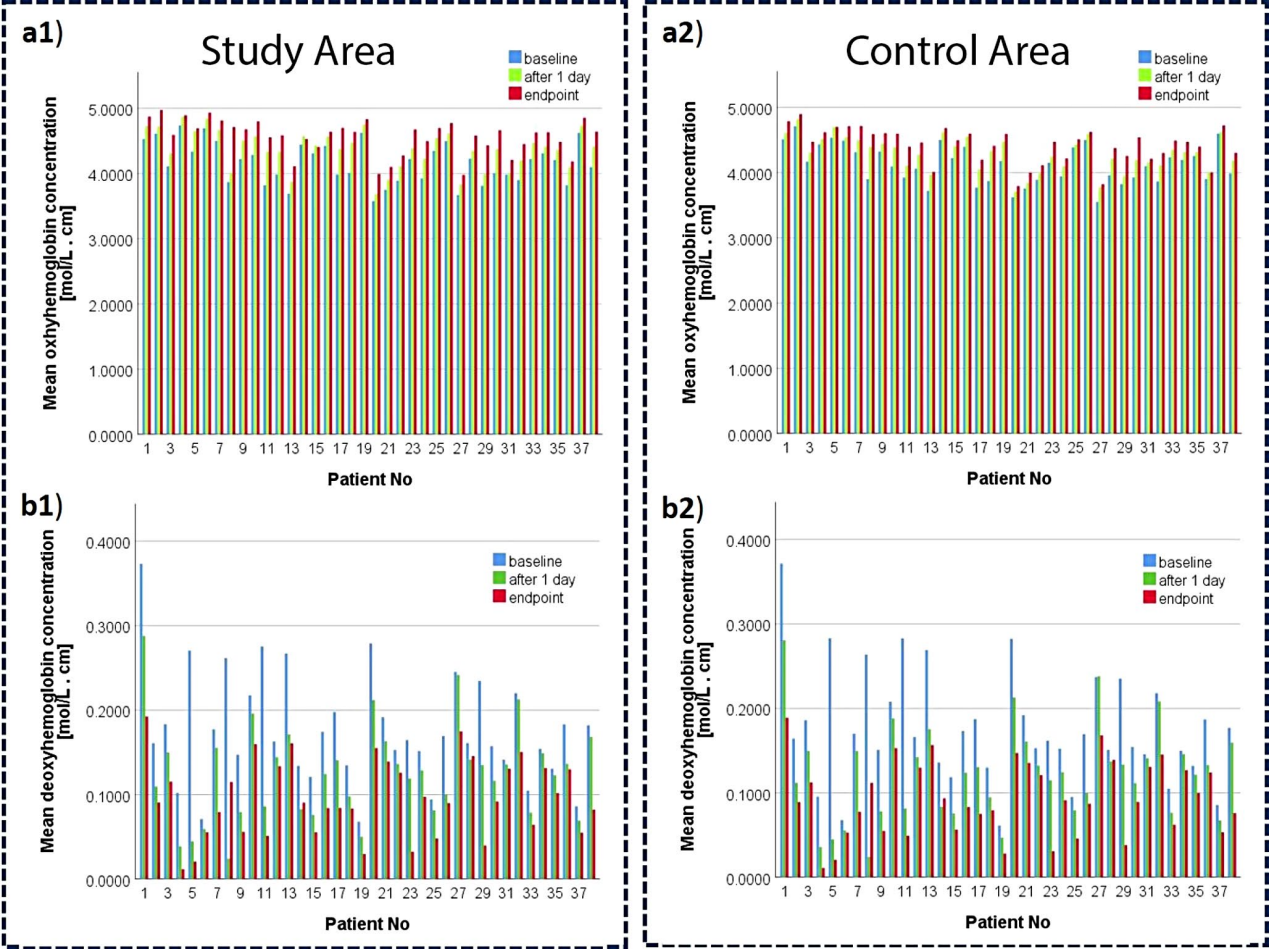
Changes in oxyhemoglobin and deoxyhemoglobin concentrations in the investigated areas of the patients (study and control areas) at different time points during mud therapy. (**a** 1) changes in oxyhemoglobin concentration in the study areas of the patients; (**a** 2) changes in oxyhemoglobin concentration in the control areas of the patients; (**b** 1) changes in deoxyhemoglobin concentration in the study areas of the patients; (**b** 2) changes in deoxyhemoglobin concentration in the control areas of the patients

As can be seen in Fig. 3, the distribution maps reveal the spatial variation of HbO_2_ and Hb concentrations as well as StO_2_ levels in the investigated areas at different time points of mud therapy, which allows an easier visual comparison of any changes that occurred within and between the study and control areas for this particular case. It is worth noting in Fig. 3b and c that there are no notable differences between the mean values of oxyhemoglobin concentrations in the study and control areas (4.0050 ± 0.1470 mol·cm/L and 3.9278 ± 0.1998 mol·cm/L, respectively) at baseline, which ensures that any post-treatment differences observed are attributable to the therapeutic intervention rather than underlying variance between the control and study areas. At the same time point, the deoxyhemoglobin concentrations recorded lower values than those of oxyhemoglobin, as expected in normal skin. The deoxyhemoglobin concentration values for this patient were in the range of (0–2) mol·cm/L for both investigated areas, with a mean value of 0.1573 ± 0.0285 mol·cm/L for the study area and 0.1543 ± 0.0192 mol·cm/L for the control area (Table 2). The calculated value of StO_2_ for this case was 96.22% indicating a normal level of tissue oxygenation at baseline.

**Table 2:** Changes in the mean values of oxyhemoglobin and deoxyhemoglobin concentrations and oxygen saturation (StO_2_) induced by mud therapy calculated for the two investigated areas (study and control areas) on the lumbar area of a 66-year-old patient diagnosed with low back pain

Time moment	Study area			Control area		
	HbO_2_ (mol·cm/L)	Hb (mol·cm/L)	StO_2_ (%)	HbO_2_ (mol·cm/L)	Hb (mol·cm/L)	StO_2_ (%)
Baseline	4.0050 ± 0.1470	0.1573 ± 0.0285	96.22	3.9278 ± 0.1998	0.1543 ± 0.0192	96.22
After 1 day	4.3783 ± 0.1023	0.1164 ± 0.0209	97.41	4.1959 ± 0.2667	0.1115 ± 0.1729	97.41
Endpoint	4.6627 ± 0.1812	0.0918 ± 0.0173	98.07	4.5409 ± 0.2549	0.0893 ± 0.1575	98.07

After the first day of treatment, as well as at the endpoint of mud therapy, an increase in the mean value of the oxyhemoglobin concentration in the study area was observed. Changes in oxyhemoglobin concentrations compared to baseline was Δ$\:{\text{C}}_{\text{H}\text{b}\text{O}2}^{\text{S}}$ = 0.3733 mol·cm/L after 1 day of treatment and Δ$\:{\text{C}}_{\text{H}\text{b}\text{O}2}^{\text{S}}$ = 0.6557 mol·cm/L at the endpoint of mud therapy. On the other hand, the concentration of deoxyhemoglobin in the study area decreased from the baseline value $\:{C}_{Hb-baseline}^{S}=$ 0.1573±0.0285 mol·cm/L to$\:\:{\text{C}}_{\text{H}\text{b}-\text{a}\text{f}\text{t}\text{e}\text{r}\:1\:\text{d}\text{a}\text{y}}^{\text{S}}$= 0.1164±0.0209 mol·cm/L after 1 day of treatment and further on to $\:{\text{C}}_{\text{H}\text{b}-\text{e}\text{n}\text{d}\text{p}\text{o}\text{i}\text{n}\text{t}}^{\text{S}}$= 0.0918±0.0173 mol·cm/L at endpoint. These findings are normal, as long as the total hemoglobin concentration remained the same throughout the study. Changes in oxyhemoglobin and deoxyhemoglobin concentrations induced in the study area during mud therapy led to an improvement in tissue oxygenation, with oxygen saturation (StO_2_) increasing from baseline to treatment endpoint from 96.22 to 98.07%.

Changes in the concentrations of oxyhemoglobin and deoxyhemoglobin during treatment were also seen in the control area. The increase in the concentration of oxyhemoglobin ($\:{\text{C}}_{\text{H}\text{b}\text{O}2}^{\text{S}}=\:$0.6131 mol·cm/L) and decrease in the concentration of deoxyhemoglobin ($\:{\text{C}}_{\text{H}\text{b}}^{\text{S}}$= -0.0585 mol·cm/L) during treatment, although smaller than in the study area. This fact is probably explained by some influence of the mud therapy in the neighboring areas, which also improved oxygen saturation.

The accuracy of determination of oxyhemoglobin and deoxyhemoglobin concentrations in skin tissue during mud therapy was evaluated based on R^2^ values. As can be seen in Fig. 3, the R^2^ values range from 0.8728 ± 0.1144 to 0.8878 ± 0.1005 (relatively close to 1), indicating that hyperspectral imaging combined with the proposed analytical model can be considered accurate enough in determining the tissue oxygenation level during mud therapy.

Changes in the oxyhemoglobin and deoxyhemoglobin concentrations in the study area of the patients (Study Area - SA), at different time points during mud therapy (baseline, after the first day of treatment and at endpoint) were significant (Fig. 4a1 and 4b1). The mean oxyhemoglobin concentration increased with$\:{\text{C}}_{\text{H}\text{b}\text{O}2}^{\text{S}}$ = 0.2127 ± 0.1096 mol cm∕L (paired-samples t test: t_37_ = 11.959, *p* < 0.05 vs. baseline) and $\:{\text{C}}_{\text{H}\text{b}\text{O}2}^{\text{S}}$ = 0.1926±0.1306 mol cm∕L (paired-samples t test: t_37_ = 9.084, *p* < 0.05 vs. after the first day). The mean deoxyhemoglobin concentration decreased with $\:{\text{C}}_{\text{H}\text{b}}^{\text{S}}$= -0.0509±0.0558 mol cm∕L (paired-samples t test: t_37_ = -5.622, *p* < 0.05 vs. baseline) and $\:{\text{C}}_{\text{H}\text{b}}^{\text{S}}$ = -0.0293±0.0341 (paired-samples t test: t_37_ = -5.303, *p* < 0.05 vs. after the first day). All these variations have statistical significance demonstrating the effectiveness of mud therapy in improving tissue oxygenation.

Figure 4.

Areas in the control area (CA) also showed statistically significant variations in oxyhemoglobin and deoxyhemoglobin concentrations (Fig. 4a2 and 4b2). The mean oxyhemoglobin concentration increased with $\:{\text{C}}_{\text{H}\text{b}\text{O}2}^{\text{S}}$ = 0.1673±0.1059 mol cm∕L (paired-samples t test: t_37_ = 9.736, *p* < 0.05 vs. baseline) and$\:{\text{C}}_{\text{H}\text{b}\text{O}2}^{\text{S}}$ = 0.1312±0.0790 mol cm∕L (paired-samples t test: t_37_ = 10.236, *p* < 0.05 vs. after 1 treatment day). The mean deoxyhemoglobin concentration decreased with $\:{\text{C}}_{\text{H}\text{b}}^{\text{S}}$= -0.0525±0.0578 mol cm∕L (paired-samples t test: t_37_ = -5.597, *p* < 0.05 vs. baseline) and$\:{\text{C}}_{\text{H}\text{b}}^{\text{S}}$ = -0.0298±0.0332 (paired-samples t test: t_37_ = -5.538, *p* < 0.05 vs. after 1 treatment day). Although these variations observed in the CA areas were statistically significant, they were in all cases smaller than in the SA areas at any time point of mud therapy.

**Fig. 5:**
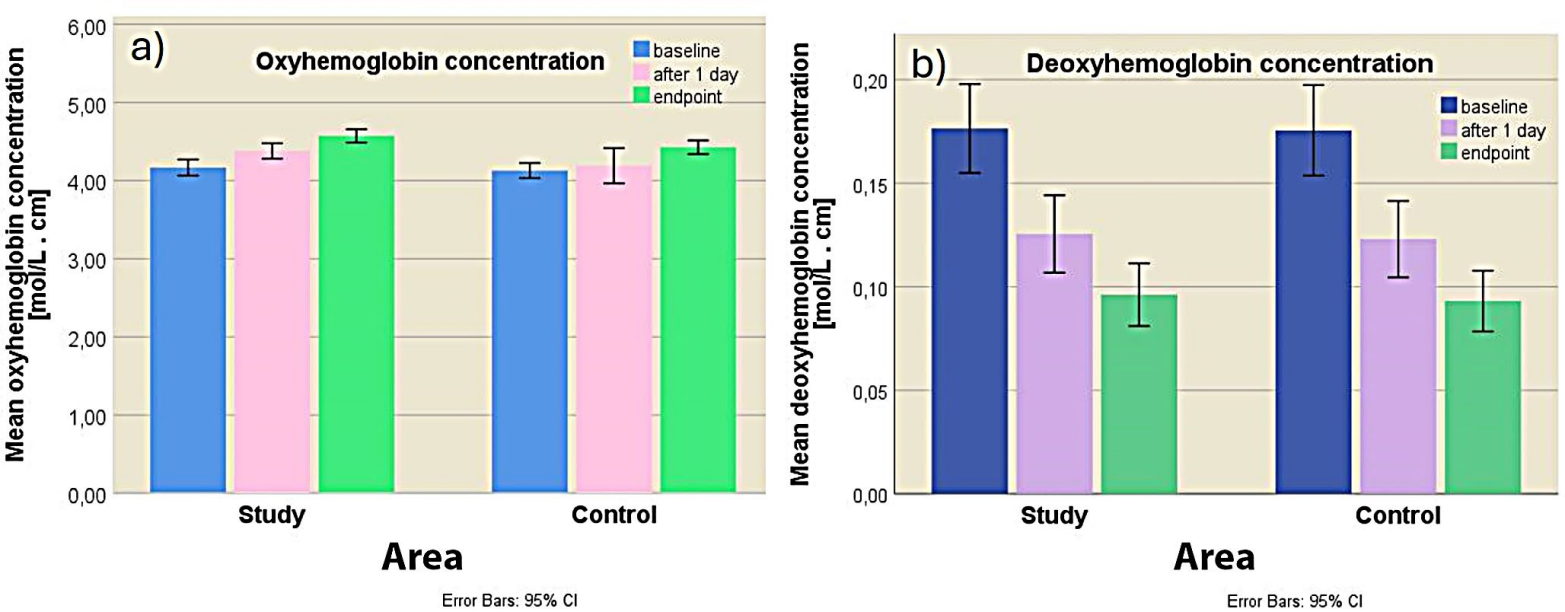
Mean oxyhemoglobin and deoxyhemoglobin concentration in the two investigated areas (study and control) calculated at different time points of the mud therapy; (**a**) mean oxyhemoglobin concentration; (**b**) mean deoxyhemoglobin concentration; (error bars show the 95% confidence interval)

A comparative analysis of variations in oxyhemoglobin and deoxyhemoglobin concentrations induced by mud therapy in study and control areas is shown in Fig. 5.

As can be seen in Fig. 5a, SA areas showed a permanent increase in mean oxyhemoglobin concentration throughout the mud therapy ($\:{\text{C}}_{\text{H}\text{b}\text{O}2}^{\text{S}}$ = 0.4053 ± 0.1787 mol cm ∕L, paired-samples t test: t_37_ = 13.983, p = < 0.05 endpoint vs. baseline) than the one recorded for the CA areas with 0.1452 ± 0.01120 mol cm ∕L ($\:{\text{C}}_{\text{H}\text{b}\text{O}2}^{\text{S}}$ = 0.2985 ± 0.1449 mol cm∕L, paired-samples t test: t_37_ = 12.698, p = < 0.05 endpoint vs. baseline). All these variations have statistical significance. The mean deoxyhemoglobin concentration decreased statistically significantly during mud therapy in both areas (Fig. 5b), slightly more in SA areas ($\:{\text{C}}_{\text{H}\text{b}}^{\text{S}}$ = -0.0802 ± 0.0573 mol cm ∕L, test pairs test t: t_37_ = 8.625, *p* < 0.05 endpoint vs. baseline) by 0.0030 ± 0.0025 mol cm ∕L than in CA areas ($\:{\text{C}}_{\text{H}\text{b}}^{\text{S}}$ = -0.8036 ± 0.0722 mol cm ∕L, test pairs test t: t_37_ = -68.527, *p* < 0.05 endpoint vs. baseline).

**Fig. 6:**
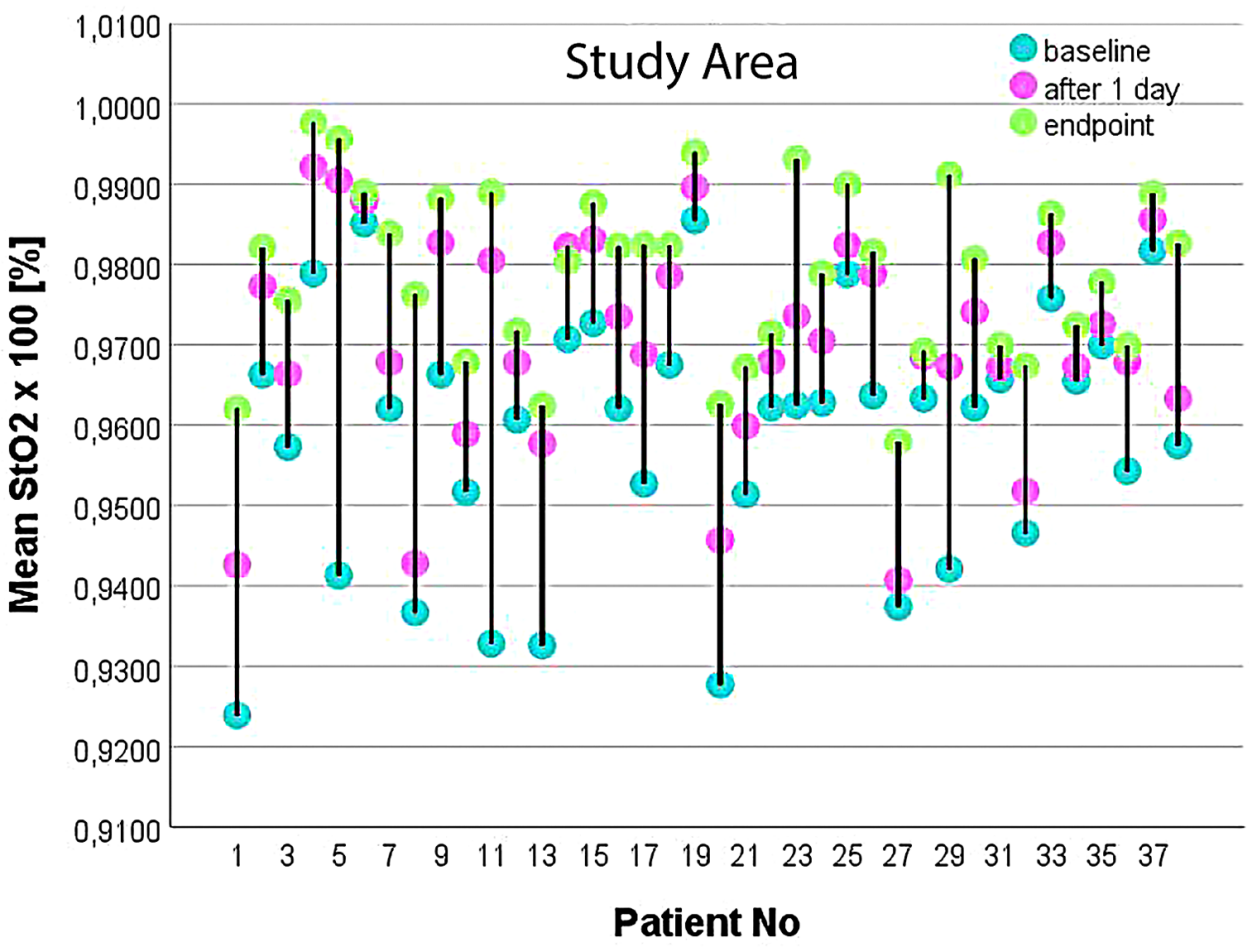
Changes in oxygen saturation values of the study area in each patient at different times during mud therapy calculated from hyperspectral data

All these changes in the concentration of oxyhemoglobin and deoxyhemoglobin are reflected in the changes in oxygen saturation values (according to Eq. 5) induced by the mud therapy (Fig. 6).

Figure 6 highlights that each patient has his own tissue oxygenation profile. Some of the patients initially showed lower oxygen saturation (e.g. patients 1, 8, 11, and 13 had a StO2 value of less than 95%) while others started mud therapy from a StO_2_ value greater than 98% (e.g. patients 6 and 19). During mud therapy, oxygen saturation increased in all patients, but at different rates. The raise was higher for patients having lower starting StO_2_ values, a fact that is logical, as long as StO_2_ has a superior physiological limit. At the end of treatment, all patients had statistically significant improvements of their local StO_2_.

These findings suggest that mud therapy can effectively enhance local tissue oxygenation in patients, potentially through mechanisms that improve blood flow, reduce inflammation, or modify other local physiological responses. The greater improvement observed in patients starting with lower StO_2_ might be indicative of the therapy’s capacity to correct more severe dysfunctions in tissue perfusion and oxygenation. Patients who showed the smallest increases in StO_2_, started with relatively high values, suggesting that individuals with less room for improvement (i.e., those starting closer to normal oxygenation levels) may experience less significant increases through mud therapy. This could be attributed to a ceiling effect where the potential for improvement is naturally limited by higher starting values. On the other hand, patients 5, 11, and 29, who had the most significant increases in StO_2_, may have started from a more compromised state of oxygenation, offering more scope for improvement. The greater initial deficit in tissue oxygenation allowed for more noticeable gains through therapeutic intervention.

The average StO_2_ values rose from a baseline of 95.8895 ± 1.5834% to 97.0488 ± 1.3513% after one day, and to 97.9156 ± 1.0421% at the endpoint. This highlights that oxygenation improves significantly in the early part of the therapy, and prolonged treatment does not result in substantial changes in the state of oxygenation. This observation might suggest that short-term interventions could be optimized for efficiency, focusing on achieving most of the therapeutic gains early in the treatment process. Exploring more in-depth variations among these themes could include investigating genetic factors, lifestyle, or specific health conditions that might influence the efficacy of mud therapy.

**Fig. 7:**
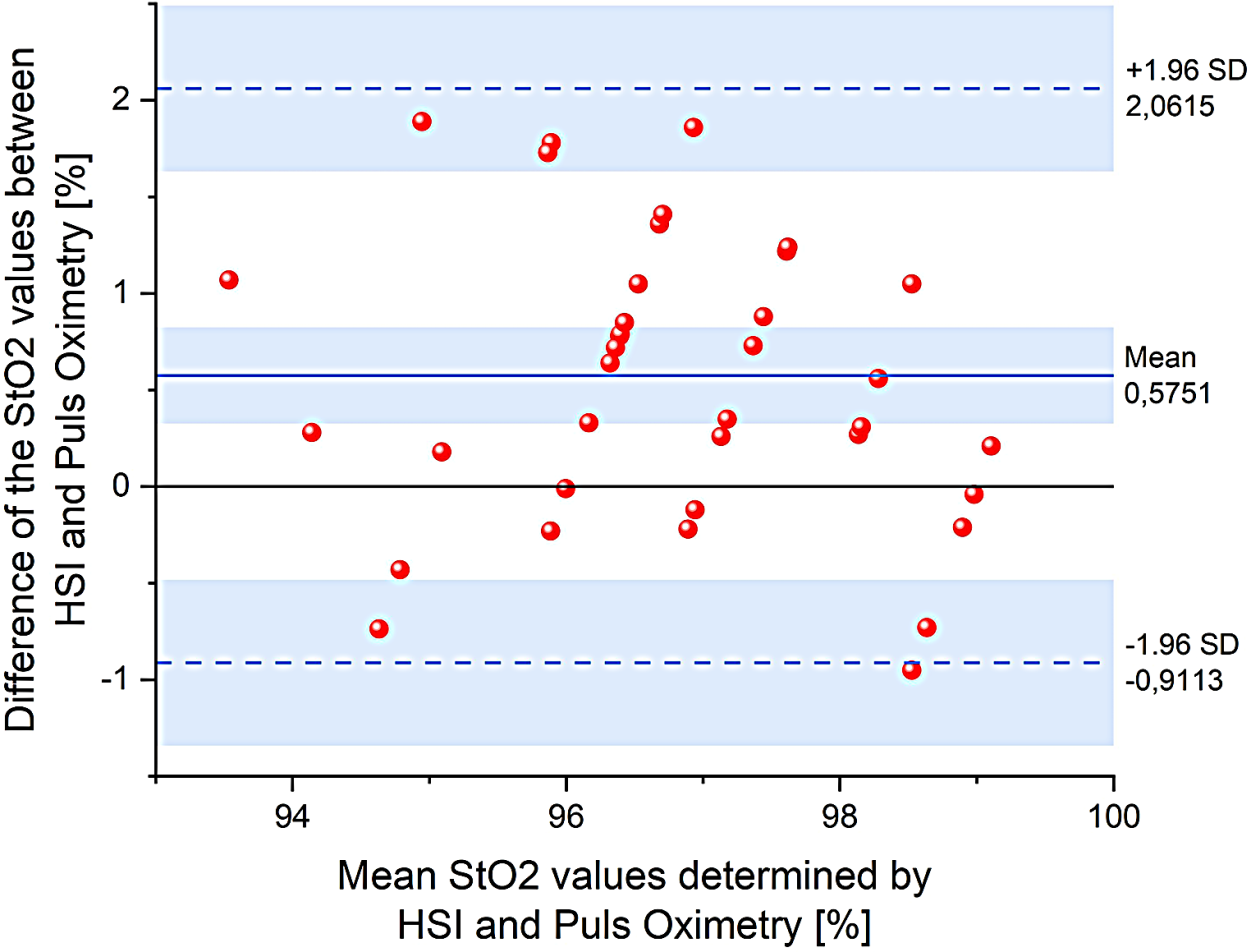
Bland-Altman plot showing agreement for measured and calculated StO_2_ values between hyperspectral imaging and pulse oximetry in the study areas at the end of mud therapy. The blue solid line indicates the mean difference (bias) between StO_2_ values determined by the two methods, and the black solid line is the line of equality. Dotted lines indicate 95% limits of agreement

The degree of agreement between oxygen saturation values (StO_2_) calculated from hyperspectral data and values measured by the widely used clinical pulse oximetry method was assessed using the Bland and Altman statistical method (Bland and Altman 1986). The differences between the StO_2_ values measured and calculated by the two methods, pulse oximetry and hyperspectral imaging respectively, plotted against the mean values according to the Bland Altman method, are shown in Fig. 7.

It is noteworthy in the Bland-Altman plot that the mean bias between the two methods is 0.5751 ± 0.1902%, and the 95% limits of agreements are narrow enough, ranging from − 0.9113 to 2.0615%. No point is found outside this confidence interval. In most cases, the StO_2_ value calculated by the HSI method is higher than that measured by pulse oximetry. This fact could be explained by the fact that the two measurements were done in different areas of the body (the lumbar area and the tip of the finger, respectively). Therefore, there is no significant difference between the two methods. We can also assume, that the values of oxyhemoglobin and deoxyhemoglobin concentration measured from hyperspectral images, which were the bases for StO_2_ estimation, are also correct (there are no other means to validate these measurements).

## Discussions

This study investigated the effects of Techirghiol sapropelic mud on tissue oxygenation in elderly patients using hyperspectral imaging method. The primary finding of the present study is that sapropelic mud, in our case Techirghiol mud, can improve local tissue oxygenation in elderly patients. Tissue oxygenation refers to the level of oxygen available in the tissues, which is critical for cellular metabolism, especially in maintaining the integrity and function of tissues. In elderly, tissue oxygenation can be reduced due to systemic (reduced cardiac output, chronic pulmonary diseases) or local factors. Since it is unlikely that pelotherapy has an influence on systemic factors when applied locally, we can assume that the noticed improved local oxygenation is due to changes in microvascular function. Our study cannot provide any clues regarding the pathophysiological paths that leads to increased tissue StO_2_, nor can discriminate between the effects of heat alone and of the mud itself. Probably, both factors have their contribution, but it was not the aim of this article to prove this. In fact, there is a scarcity of publications that explain the mechanisms producing the benefactory effects of peloids, as pointed up recently by Carretero (Carretero 2020). Studies have shown that heat alone can promote vasodilation, increasing local blood flow and oxygenation (Kellogg et al. 2006). However, the chemical composition of sapropelic mud, rich in minerals such as sulfur, magnesium, and calcium, may have additional anti-inflammatory and vasodilatory effects, further enhancing tissue oxygenation (Fioravanti et al. 2011; Gomes et al. 2013). It is, however, worth pointing out that the increases were observed in all the patients in the group, irrespective of the illnesses that they were treated for. Another important finding was that the increase in local tissue oxygenation was noted after the first day of treatment, although it slowly continued throughout the study, but with a slower rate. This suggests that short term applications may have a significant effect when it comes to improving skin and subcutaneous tissue StO_2_. It is also important to point out that the increase of tissue oxygenation was significantly greater in the treated areas compared to control zones, but some improvement was noted even in controls, probably due to a neighboring effect. The mud was not applicated over the patch to avoid any permeability issues while the location of the patch was consistent in all cases. While other explanations for the response of the control areas might exist, our study could not identify or prove them.

Our findings align with those from Poensin et al. ​​(Poensin et al. 2003), Clijsen et al. (Clijsen et al. 2008)​​, Kasimova et al. (Kasimova et al. 2017), and Tuulik et al. (Tuulik et al. 2015), who also reported similar improvements in microcirculation and tissue oxygenation after mud therapy. Marin et al. (Marin et al. 2011, 2012) reported improvement of partial pressures of blood gases after applications of the same Techirghiol mud, results that are similar to ours.

The present study also demonstrates that hyperspectral imaging combined with the analytical model described for the generation of the oxyhemoglobin and deoxyhemoglobin concentrations distributions maps is able to highlight and monitor the changes that mud therapy is inducing in the tissue oxygenation.

Other investigators (Poensin et al. 2003; Clijsen et al. 2008) used Doppler ultrasonography to demonstrate improvements of blood flow following mud therapy. Hyperspectral imaging is, thus, a relatively simple, easy to use and non-invasive method for spectral characterization of skin and subcutaneous tissue.

The increase in tissue oxygenation in response to Techirghiol mud therapy suggests potential clinical applications, particularly in the management of conditions characterized by poor microcirculation and oxygenation such as diabetic ulcers, peripheral arterial disease, and various age-related circulatory issues. The ability to monitor changes in chromophore concentrations through hyperspectral imaging could aid clinicians in tailoring treatments to individual patient needs, optimizing therapeutic outcomes.

While the findings are promising, the study has limitations that must be acknowledged. The study design (non-randomized) and the small sample size are the main limitations, leading to a lower power of the conclusions, but they do not change the statistical significance, especially with the self-control design which partially compensates the non-randomization. The study was restricted to a single geographical location. Future research should aim to replicate these findings in a larger cohort and diverse settings. Additionally, the study demonstrated an increase in tissue StO_2_ only for one week, during pelotherapy. Long term follow ups are needed in order to prove that this therapeutic effect persists after the therapy ceases and lasts long enough to produce reliable results. It should be noted that the study demonstrated an increase in oxygenation in an area and in patients without proven local circulation insufficiency. It would be interesting to test these effects, monitored with hyperspectral imaging technology, in patients with documented peripheral vascular disease.

Future research should aim to isolate the specific therapeutic effects of mud versus the effects of heat. This could be achieved by conducting randomized controlled trials in which one group receives only heat therapy, while the other receives both heat and mud therapy. Such studies would provide clearer insights into the relative contributions of these factors. Additionally, we recommend expanding this research to include patients with conditions such as peripheral vascular disease, where improving local circulation is particularly relevant. Studying this population could help further elucidate the therapeutic potential of Techirghiol mud in managing diseases characterized by poor microcirculation. In terms of methodology, future studies could benefit from using multi-modal imaging techniques, such as a combination of hyperspectral imaging and laser-Doppler flowmetry, to more comprehensively assess changes in tissue oxygenation and microcirculation. Increasing the sample size and conducting the studies in a controlled clinical setting with a larger and more diverse patient population would also strengthen the findings and enhance the generalizability of the results.

## Conclusion

This study underscores the potential of integrating traditional natural resources sapropelic mud from Lake Techirghiol in enhancing tissue oxygenation in elderly patients, with hyperspectral imaging providing a valuable and reliable tool for monitoring therapeutic effects.

By validating traditional therapies and providing scientific evidence of the benefits of mud therapy, our study supports the integration of traditional natural treatments into modern medical paradigms.

## Data Availability

In accordance with the requirements of the General Data Protection Regulation (GDPR) (EU) 2016/679, the data (patient images) cannot be made available.
